# Supplementary material for: Association between Visceral Adiposity Index and Hyperuricemia among Steelworkers: The Moderating Effects of Drinking Tea
Source: Nutrients. 2024 Sep 23;16(18):3221. doi: 10.3390/nu16183221 (PMC11435409; doi:10.3390/nu16183221)
Supplement: Supplementary file 1 [file nutrients-16-03221-s001.zip › nutrients-3202861-supplementary.pdf]

Attachment Supplementary Table S1 VAI and uric acid levels in populations with different characteristics (*n* = 9928).

Variables	n (%)	VAI (Median (IQR))	*p*	Uric acid (mean ± SD)	*p*	Hyperuricemia (n, %)	*p*
Age							
<45	4706(47.40)	2.15(1.22-3.71)	<0.001	6.02 ± 1.46	<0.001	1213(25.78)	<0.001
≥45	5222(52.60)	2.22(1.31-3.82)		5.90 ± 1.37		1144(21.91)	
Sex							
male	8159(82.18)	2.37(1.41-4.06)	<0.001	6.16 ± 1.35	<0.001	1992(24.41)	<0.001
female	1769(17.82)	1.45(0.84-2.46)		5.00 ± 1.30		365(20.63)	
Smoking							
yes	5731(57.73)	2.04(1.19-3.57)	0.970	5.98 ± 1.42	0.056	1393(24.31)	0.122
no	4197(42.27)	2.06(1.19-3.48)		5.92 ± 1.39		964(22.97)	
Drinking alcohol							
yes	5265(53.03)	2.06(1.21-3.53)	0.268	5.99 ± 1.42	0.020	1258(23.89)	0.704
no	4663(46.97)	2.03(1.17-3.53)		5.92 ± 1.40		1099(23.57)	
Physical activity							
yes	2609(26.28)	2.02(1.16-3.44)	0.179	5.91 ± 1.39	0.052	599(22.96)	0.274
no	7319(73.72)	2.05(1.20-3.55)		5.97 ± 1.42		1758(24.02)	
Smoked food							
yes	7392(74.46)	2.05(1.20-3.54)	0.299	5.96 ± 1.41	0.424	1774(24.00)	0.302
no	2536(25.54)	2.04(1.17-3.50)		5.94 ± 1.12		583(22.99)	
Pickled food							
yes	6207(62.53)	2.04(1.19-3.49)	0.896	5.97 ± 1.42	0.249	1491(24.02)	0.397
no	3721(37.47)	2.05(1.18-3.62)		5.94 ± 1.40		866(23.28)	
Drinking tea							
yes	5704(57.45)	2.03(1.17-3.56)	0.305	5.95 ± 1.42	0.892	1385(24.28)	0.142
no	4224(42.55)	2.08(1.21-3.49)		5.96 ± 1.40		972(23.01)	
Occupational exposure							
yes	3505(35.30)	2.15(1.25-3.65)	<0.001	5.97 ± 1.39	0.495	881(25.13)	0.016
no	6423(64.70)	2.00(1.15-3.46)		5.95 ± 1.42		1476(22.98)	
Hypertension							
yes	1351(13.61)	2.53(1.47-4.17)	<0.001	6.29 ± 1.45	<0.001	418(30.94)	<0.001
no	8577(86.39)	1.98(1.15-3.42)		5.90 ± 1.40		1939(22.61)	
Diabetes							
yes	651(6.56)	3.19(1.97-5.66)	<0.001	5.87 ± 1.38	0.118	122(18.74)	0.002
no	9277(93.44)	1.98(1.16-3.38)		5.96 ± 1.41		2235(24.09)	
